# Variant Anatomy and Its Terminology

**DOI:** 10.3390/medicina56120713

**Published:** 2020-12-18

**Authors:** David Kachlík, Ivan Varga, Václav Báča, Vladimír Musil

**Affiliations:** 1 Department of Anatomy, Second Faculty of Medicine, Charles University, V Úvalu 84, 15006 Prague, Czech Republic; david.kachlik@lfmotol.cuni.cz; 2 Department of Health Care Studies, College of Polytechnics Jihlava, Tolstého 16, 58601 Jihlava, Czech Republic; vaclav.baca@vspj.cz; 3 Institute of Histology and Embryology, Faculty of Medicine, Comenius University in Bratislava, 81372 Bratislava, Slovakia; 4 Centre of Scientific Information, Third Faculty of Medicine, Charles University, Ruská 87, 10000 Prague, Czech Republic; vladimir.musil@lf3.cuni.cz

**Keywords:** anatomy, anatomical variations, variability, terminology, nomenclature

## Abstract

Variant anatomy, which is an integral part of anatomical science, is related to abnormalities in the human body structure. Our understanding of variant anatomy is based on thousand years of anatomical experience. These abnormalities generally do not interfere with the function of the human body and do not typically manifest as pathological nosological units. However, under certain conditions, these abnormalities can worsen existing pathological states or even evoke new ones. Understanding variant anatomy is a basic skill not only of mere anatomists, but also of clinicians who work in fields involving both diagnostic techniques and therapeutic interventions. To gain and retain a good knowledge of the most frequent and clinically relevant anatomical variations, a simple, clear, and exactly defined nomenclature of variant structures is needed. A list of items comprising variant anatomy, which have been incorporated into the internationally accepted nomenclatures *Terminologia Anatomica* (1998) and *Terminologia Neuroanatomica* (2017), is described and analyzed. Examples of the most common anatomical variations related to terminology are mentioned, and variant anatomy as a whole and its role in understanding current anatomy are discussed.

## 1. Introduction

*Motto: “Variability is the law of life“—Sir William Osler*

The human body is a very complicated and sophisticated unit that develops from two single cells that merge and subsequently divide into 37.2 ± 0.81 trillion cells [1]. During development, divergence from the DNA-encoded body plan happens, which on one side allows further improvements, but on the other side can lead to developmental defects or death via spontaneous abortion. If anatomical divergence from the norm does not affect function, it is not considered a developmental defect (congenital malformation) but, rather, a mere anatomical variation.

Variant anatomy is a field of anatomical science that concerns the abnormalities of the human body structure. What is considered normal with regard to the human body structure is arbitrary and it is based on thousands of years of experience and/or agreements among specialists and their societies and committees. In case of variant anatomy, these abnormalities do not typically interfere with the function of a given part of the human body and, thus, do not usually manifest as pathological nosological units. Without a doubt, these abnormalities should be congenital/development defects, but generally they are not considered defects due to the above-mentioned fact that they do not usually have pathological manifestations.

However, under any changed condition (e.g., ischemia, malnutrition), such variants either can remain silent both functionally and clinically or can worsen the pathological state or even evoke a new one. The presence of an anatomical variant can be revealed during a diagnostic or therapeutic intervention and can be an unpleasant surprise for the intervening specialist. For these reasons, it is necessary to consider variant anatomy an unavoidable aspect of the human body, equally important to the clinician as systemic and topographical anatomy.

The aim of this review is to discuss the general terminology concerning anatomical variants, define basic terms, raise questions about the frequency and reliability of the reported numbers of anatomical variants in the human population, refer to their clinical relevance, and emphasize the terminology in this field as it is the most neglected aspect of anatomical variations. The last task is to review the current terminology surrounding norms and present examples from vascular anatomy (the most variable system of the human body) as well the other organ systems. These examples are based on and selected in relation to the authors’ own experience, findings, and publications.

First, it is important to review key principles related to the construction of the human body [2] and to define principal terms based on the British Oxford English Dictionary [3] and the American Merriam-Webster Dictionnary [4]:

- normal (from Latin *norma*) = conforming to standard, regular, usual, typical [3]; conforming to a type, standard, or regular pattern: characterized by that which is considered usual, typical, or routine; according with, constituting, or not deviating from a norm, rule, procedure, or principle; occurring naturally; not exhibiting defect or irregularity [4];
- abnormal (from Latin *abnormis*) = exceptional, irregular, deviating from type [3]; deviating from the normal or average [4];
- anormal = not normal—used in distinction from the positive emphasis of abnormal [4]; not listed in [3];
- anomalous (from Greek ἀνώμᾰλος) = irregular, abnormal [3]; inconsistent with or deviating from what is usual, normal, or expected; of uncertain nature or classification [4];
- variant (from Latin *variare*) = differing in form or in details from the one named or considered, differing thus, among themselves [3]; manifesting variety, deviation, or disagreement; varying usually slightly from the standard form [4];
- variable = that can be varied or adapted; apt to vary, not constant, fickle, unsteady; including individuals or groups that depart from the type; tending to change in structure or function [3]; able or apt to vary: subject to variation or changes; characterized by variations; not true to type [4];
- accessory (from Latin *accessus*) = additional, subordinately contributing, dispensable, adventitious [3]; present in a minor amount and not essential as a constituent; assisting under the orders of another; aiding or contributing in a secondary way [4];
- aberrant (from Latin *aberro*) = diverging from the normal type [3]; deviating from the usual or natural type; straying from the right or normal way [4];
- typical (from Greek τύπος) = serving as a type or characteristic example, representative, symbolical, emblematic [3]; combining or exhibiting the essential characteristics of a group; conforming to a type; constituting or having the nature of a type [4];
- atypical = non-conforming to a type [3]; not typical [4];
- constant (from Latin *constare*) = unmoved, resolute, faithful, unchanging, unremitting, frequently occurring [3]; marked by firm/steady/fast resolution or faithfulness: exhibiting constancy of mind or attachment; invariable, uniform; continually occurring or recurring (regular) [4];
- inconstant = fickle, changeable, variable [3]; likely to change frequently without apparent or cogent reason [4].

According to Keith Moore [5], the term normal means “within the normal range of variation.” The Latin word *normalis* means conforming to the rule or pattern. The English word normality denotes the state of being normal, and the verb normalize means to make normal. It is used in descriptive anatomy to indicate the standard or normal appearance of a structure [5]. Additionally, the term *norma* is used in *Terminologia Anatomica* five times to describe different views of the typical appearance of the skull only (*norma facialis*, *superior*, *occipitalis*, *lateralis*, *inferior*) and is synonymous with the English term aspect. R. B. Salter goes further with his statement: “While one speaks of an average infant, an average child and an average adult, it is important to appreciate that there exists an extremely wide range of normal in body form and function” [6].

The most common type of structure is the one best fitted for survival. However, frequency alone is not the criterion for normality [5]. The overall prevalence of an arrangement of a specific structure in the human population is the only decisive criterion for determining if a structure is constant or variant. At what level does one consider a structure to be variant? This point has not yet been agreed upon in this field and remains an unsolved task for experts in the future. Keith Moore stated in 1989 that any feature, such as the protuberance of a bone or the branching of an artery, should be called normal if it falls within the normal range of variation. However, many textbooks and lecturers fail to explain in their introductions that this is what they mean when they use the term normal [5]. Almost any minor variation may be observed as a normal feature for a particular family; however, finding several minor variations in the same individual is unusual, and textbooks and lecturers may therefore indicate that a serious malformation (e.g., of the cardiovascular system) has occurred during development in these cases [5,7].

Anatomical variations are not only very interesting and thought-provoking for anatomists but also of principal concern for clinicians. Theodore E. Keats stated in his editorial that “I have spent a large portion of my professional life collecting and documenting normal anatomical variants, and I find the task endless. It seems that Mother Nature is boundless in the infinite variations in the way we are constructed.” Keats has also argued that “the interpretation of normal variants must always include proper incorporation with clinical history and physical findings, or we may inadvertently give patients diseases which they do not have, and this is probably the worst mistake we can make in medicine” [8]. Cahill and Leonard stated in 1999 that 10% of surgical errors in the USA are due to the lack of knowledge of existing anatomic variations [9]. The need for precise knowledge of human anatomy has to be emphasized, especially in the technical fields of medicine (ultrasonography, magnetic resonance, computed tomography, endoscopy, surgery, etc.) in which this knowledge can lead to a better and more accurate diagnosis, help prevent mistaking a variation for a pathology, as well as allow better therapeutic interventions [10]. That is why expert educational committees in the USA and the Netherlands have included anatomical variations in their anatomy programs, objectives, and syllabi [11,12].

## 2. Variation as a Unit and as a Part of the Whole

Across the human population, the body displays a certain degree of differences with regard to its volume, shape, structure, position, etc. Morphological fluctuations in organs or other parts of the body that do not result in a functional “handicap” for the individual may be considered an “anatomical variation” [13].

Many different types of anatomical variants exist, such as supernumerary bony structures, missing or supernumerary muscle heads and bellies, changes in the shape and position of organs and their parts, supernumerary (accessory), missing, aberrant, and rudimentary vessels, and differing numbers of lymph nodes. Even the atavisms and rudiments cannot be omitted, which are known from veterinary and comparative anatomy (hairs, muscle bundles, *tuberculum auriculare* of Darwini of the auricle, etc.). These structures are common in animals, but it is rare to find them in humans. When found, these abnormalities usually do not impact an individual’s physiology. They can be termed “normal variations” [14]. On the other hand, all morphological fluctuations that are beyond the mentioned limits of normality are defined as anomalies or malformations. These abnormalities constitute a true functional handicap for the individual [14]. Anomalies or malformations are considered synonyms for structural abnormalities [15]. The causes of variations and anomalies are rooted in biological processes during development, including the formation of a particular structure. This may be due to genetic or environmental reasons or their combination [14].

Based on the above examples, it is obvious that there are many structures within the scope of variant anatomy. Singer concluded in 1956 that “to recognize anatomical variations, it is necessary to previously establish the normal pattern of the human body and name its structures.” Galen initiated this effort centuries before Vesalius, based on his clinical practice and dissection of apes, dogs, and other mammals [16].

Who was the first to describe a variant anatomical structure and who was the first to define a prevalence for a specific variant? These questions require thorough historical exploration. We can see many variations in Andreas Vesalius’ *De Humani Corporis Fabrica* published in 1543, which is surely based not only on his own observation, but also on the sources of previous Greek, Latin, and Arabic authors [10,17,18]. Scientists are still working on recognizing and classifying anatomical variations. It is not always easy to define variations precisely, because inexact descriptions and incorrect terminology related to anatomical variations often lead to errors, mismatches, and mistakes when determining the statistical frequency of variations.

Modern definitions describe an anatomical variant as “a deviation from the ‘normal’ arrangement of an anatomical structure without causing a demonstrable impairment to its functioning” [19,20]. This is in contrast to a pathological abnormality, which may impair functioning of one or more anatomical structures. While many anatomical variants do not require clinical attention, some may present diagnostic problems or cause adverse symptoms [21].

As for frequency, variations can be classified as frequent, infrequent, rare, or sporadic. Frequent variations can even reach 100% in case occurrence. In this case, the intended structure is either present persistently or absent in a few percent of cases (e.g., *ossa suturalia* are found in nearly all skulls). Otherwise, the variation frequency equals half of the cases at most (e.g., the position of the *arteria maxillaris* in relation to the *musculus pterygoideus lateralis* in the *fossa infratemporalis* is, according to many studies, medial to the artery position in approximately in 30–45% of cases and lateral in 55–70% of cases [22]). Infrequent variations are present in 1–10% of cases (e.g., *arteria thyroidea ima* is reported to appear in approximately 0.4–10% of cases [22]), and rare variations are present in less than 1% of cases (e.g., *situs viscerum inversus*). Variations are considered sporadic if the number of total cases reported does not exceed 100 (e.g., *arteria mesenterica media*, which has only been reported in a total of 17 cases according to our own findings [23]). Studies of variant anatomy performed in recent decades have often uncovered new, surprising findings that sometimes contradict textbook norms, especially thanks to new scientific approaches and techniques (e.g., angiography, CT angiography, 3D recontructions, CT microangiography, duplex ultrasonography, endoscopy, etc).

Variant anatomy is also important because it has clinical relevance. If we revisit the previously mentioned examples of variations, we can conclude that the positional relationship between the *arteria maxillaris* and the *musculus pterygoideus lateralis* is critical to know during very difficult interventions occurring in the tissue under the *ramus mandibulae*, which is not a common surgical procedure. In contrast, the *arteria thyroidea ima*, which is a much rarer variation, can cause considerable bleeding during a much more common procedure, inferior tracheotomy, due to the course of the *arteria thyroidea ima* ascending in front of the trachea in the *spatium suprasternale* towards the thyroid gland. The *situs viscerum inversus* can cause diagnostic and therapeutic problems, and the *arteria mesenterica media*, if ligated during surgical procedures (e.g., para-aortal lymphadenectomy or abdominal aorta aneurysm repair), can be a cause of ischemia and possible necrosis of variably long segments of the large intestine. Namely, abdominal aneurysm procedures and the preceding angiographies have revealed several new cases of this variant, which had been previously considered unique. It can be concluded that the real frequency of this variation is higher than we originally supposed, and extensive studies concerning variant anatomy have an important place in medicine.

It is worth mentioning that the classification of variations can be single or associated. The typical associations of variations are considered nosological units (syndromes such as Fallot’s trilogy, tetralogy, or pentalogy) but are defined as pathological units. Variations are developmental defects, anomalies, or malformations, which Arey [14] classified in minor and major anomalies. Children with only one minor anomaly have a 3% risk of developing a major defect. However, children with two minor anomalies have a 10% risk of developing major anomalies. Finally, those children with three or more minor anomalies have a 20% risk of developing a major anomaly [24,25]. The incidence of minor anomalies is reported as being between 7% and 41%, while the incidence of major anomalies is 2–3% [26]. Arey [14] concluded that the presence of a minor anomaly must alert us of the possibility of finding a major defect, while the existence of an anatomical variation does not.

Our understanding of variant anatomy is developing and changing, not only thanks to new diagnostic methods (CT, MRI, ultrasonography), but also by means of old and well-established methods (dissection, injection, corrosion casting). For example, a study performed by Andreo et al. [27] called “Caroticotympanic Artery: Anatomically a Normal Variation of the Internal Carotid Artery?” concludes that “anatomically, the artery should be considered as a variation and not as a normal branch of the petrous portion of the internal carotid artery” because it was found only in 1 out of 40 specimens (i.e., in 2.5% of cases).

Although variant anatomy is a vast topic, comprehensive sources exist. Many classical anatomical atlases contain paragraphs or pages on anatomical variants, usually concluding relevant chapters. Specific monographs on variant anatomy of the skull [28,29], upper limb muscles [30], arterial [13,31] and venous [32,33] circulatory system, and peripheral nerves [34] are available, but there also exist single monographs [22,35] and a website [36] describing the variants of the whole human body.

## 3. Variation Terminology

With regard to the clinical relevance of some anatomical variants, variant terminology not only is a part of anatomical terminology, but also has “infected” the anatomical nomenclature. As early as 1895, the *Basiliensia Nomina Anatomica*, containing 4311 items, featured 86 terms for variant structures (1.99%). The *Ienaiensia Nomina Anatomica*, with 4329 terms, included only 76 items related to variant anatomy (1.76%). The *Parisiensia Nomina Anatomica*, with 4822 terms, included only 89 items (1.85%). The current version, *Terminologia Anatomica* (TA), with 7635 terms, altogether had only 149 items for variant anatomy (1.95%) [37]. Finally, the *Terminologia Neuroanatomica* (TNA) from 2017, with 4413 terms, contains only 41 variant items (0.93%) [38]. This suggests the very erroneous idea that there exist just a few anatomical variants in the human body, and it is necessary to correct this misconception as it can have dangerous consequences in clinical medicine.

A systemic variant anatomical nomenclature does not exist, and establishing it is a task for future anatomists and the International Federation of Association of Anatomists (IFAA). Tables S1 and S2 summarize all variations explicitly listed in the last revision of anatomical nomenclatures [39,40]. This included 149/41 items across nearly all chapters, but most variants related to the cardiovascular system, which included 21% of all variants (31 items). In TA/TNA, these items are described as “normal” items, including a unique identification number (not in TNA), the official Latin term, and the most common English equivalents. However, they are distinguished by closed parentheses.

All of these terms can be viewed from different perspectives. For example, the osseous structures, such as the *ossa suturalia*, *tuberositas masseterica*, or *tuberositas pterygoidea*, are almost always described as variants, while a very frequent osseous bridge dividing the *foramen transversarium* into two unequal openings (the larger one for the *arteria vertebralis* and the smaller one for the *vena vertebralis*) has been omitted in the TA.

Eponyms are very common types of variant anatomy because such structures were initially described a long time ago, were forgotten, and then were rediscovered by later anatomists when reading these reports. Thus, these variants have been ascribed several systemic and eponymic definitions. The *musculus sphincter ductus choledochi* (called the Oddi’s or Glisson’s sphincter) or the *ligamentum inguinale* (termed Poupart’s, Vesalius’ or Falloppio’s ligament) are examples of normal anatomy. The *arteria thoracica superior* (or *arteria thoracica suprema*, *arteria thoracica superficialis*, Haller’s artery, Pelegrin’s artery) used to be considered a variant, but the TA now lists it as a norm. Finally, there are structures described only with an eponym without an appropriate Latin term. For example, Zuckerkandl’s tubercle of the thyroid gland [41], Eisler’s pocket or fat pad of the lower eyelid [42], Bochdalek’s or Foltz’s valve at the beginning of the *canaliculus lacrimalis*, Vater’s duct, His’ duct, or Bochdalek’s duct are all terms denoting one of the cavities connected with the embryonic *ductus thyroglossus* [41,43,44,45,46,47,48]. Riche-Cannieu anastomosis between the *ramus profundus nervi ulnaris* and the *ramus recurrens nervi medianus* in the hand [49], Kaplan’s accessory branch from *ramus cutaneus dorsalis nervi ulnaris* arising proximal to the *processus styloideus ulnae* and connecting the back to the nerve trunk [50] are also examples. However, attention must be paid when an eponym describes more structures, such as Müller’s muscle, which is used to describe the *fibrae circulares musculi ciliaris*, *musculus orbitalis*, and *musculus tarsalis superior* [43,44,45,46,47]. The use of eponyms is in sharp contradiction to the recommendation of the IFAA to omit all eponyms in the description of anatomical, histological, and embryological structures (with the exception of a few generalized terms summarized in Table S3). The recommendation is then to create Latin and English nomenclatures for basic and clinically relevant anatomical variations, which can be successively completed and changed according to the needs of both anatomists and clinicians. Except for the above-mentioned 149 items listed in the TA (Table S1), there are many other variations. These variations are of important clinical relevance because the terminology is confusing and multi-synonymous. It is necessary to simplify and classify these mistakes in terminology and remove all eponyms from variant terminology, which should be replaced with systemic Latin and English terms as recommended by the IFAA. Although eponyms are clinically widespread and enhance communication, they are neither systemic nor transparent. Their successive replacement will be painful for older generations of clinicians, but for the next generation, it will be a relief. The best platforms for discussing these changes are anatomical and clinical scientific journals and the internet.

## 4. Vascular Variations

The most variable part of the human body is the cardiovascular system, especially veins. Their arrangement, mainly in the limbs, is very heterogeneous, although they follow a certain principal axial pattern. The nomenclature of the lower limb veins and some other veins of the pelvis has been substantially expanded from 2001 to 2004 [51,52,53,54,55,56]. As for the arterial system, it has the best-described nomenclature and is recognized as part of variant anatomy. The basic and, until now, matchless publication on this topic is “Arteriensystem der Japaner” by Adachi [31]. Much less particular, but more comprehensive and general, are “Arterial Variations in Man: Classification and Frequency” [13] and the radiological “Atlas of Normal and Variant Angiographic Anatomy” [57]. The most recent publication on this topic is the “Bergman’s Comprehensive Encyclopedia of Human Anatomic Variation” [22]. In individual areas, the most impressive are the works of Michels concerning the blood supply of the unpaired abdominal organs [58,59,60,61,62], which have been extended and upgraded by our team [63]. Rodríguez-Niedenführ’s analysis of the upper limb arterial variations [64,65,66,67,68] is also a stellar study, and as for the veins, Gillot and Uhl’s studies of lower limb veins have to be mentioned [69,70,71,72,73,74,75,76]. Classical works also cannot be ommited, and we should emphasize Quain’s “Anatomy of the Arteries of the Human Body” [77] and Dubreuil-Chambardel’s monographs on the upper and lower limb arteries in French [78,79].

Clinically, the most important variants are those of the brain and heart circulation and those of the aortic arch. Students must be taught that there is much variation in the size of the component arteries of the arterial circle. One or more of the communicating arteries is often absent or is so small as to have cerebral function [80]. The *circulus arterious cerebri* (of Willis) is macroscopically present in approximately 90% of cases, but a properly developed and symmetrical circle is found in less than 50% of individuals [81]. In 60% of cases, at least one component of the *circulus arterious cerebri* is relatively hypoplastic, and, thus, the capacity to provide collateral flow is reduced [82]. The arrangement of the branches of the *arcus aortae* that is considered normal is encountered in only 65% of cases [80]. The number of aortic arch branches is reduced to two in 27% of cases, and both *arteriae carotides communes* can arise from the *truncus brachiocephalicus* (obsoletely called “*arteria innominate*”). The most common variant of the arcus aortae is called bovine arch (*arcus bovinus*) due to its similarity to the branching pattern of the *arcus aortae* in bovines, but this term is not systematic and instructive (*truncus brachiobicephalicus* or *truncus brachiobicaroticus* are possibly better terms). There exist several other less common variations of the aortic arch branches [83], which are presented by Moore as normal variations. However, one of these, the *arteria subclavia aberrans retrooesophagea* (obsoletely called “*arteria lusoria*”) arising as the last branch from the arcus aortae and passing posterior to the trachea and oesophagus, is present in 0.5–2% of cases [84] and is often combined with the non-recurrent laryngeal nerve [85,86,87]. Moore considered it to be an abnormal variation, meaning “not within the normal range of variation and, therefore, a congenital malformation” [5].

Variant anatomy includes not only life-threatening variants, but also other clinically important ones. The above-mentioned Rodríguez-Niedenführ’s analyses of the upper limb arterial variations [65,66] clarifies some potential for failure of catheterization due to a patient’s anatomical variation when performed via the *arteria radialis*. Thus, knowledge of these anatomical variations is crucial for interventional cardiologists. Our group can present another example that is important for plastic and reconstructive surgeons, concerning the blood supply of the thumb. Based on our review, the principal arterial source for the thumb, the *arteria princeps pollicis* branching from the *arteria radialis* and corresponding to the *arteria metacarpalis palmaris prima*, is the dominant source of blood supply for the thumb in only 64.8% of cases. In 85.8% of cases, one artery supplying the thumb has a distinctly larger diameter than others. The current terminology is not specific enough, and, therefore, we suggest avoiding the term “*arteria princeps pollicis*”, which evokes exceptionality and dominance in the thumb’s blood supply, and refer to all the arteries by their systemic anatomy [88].

Moreover, some existing anatomical variants of potential clinical interest have been highlighted and studied in detail, and nomenclature has been proposed, such as accessory artery braching from the *arteria radialis* that runs on the dorsal surface of the *musculus interosseus dorsalis primus* across the first web space in about 12% of cases (with the proposed name *ramus dorsalis superficialis arteriae radialis*) [89]. Thanks to its large caliber and terminal anastomosis with the *arcus palmaris superficialis*, it can serve as a favorable pedicle for cutaneous and fasciocutaneous flaps [90].

The accumulation of several arterial variations in one individual can also appear as a result of random development. However, attention must be paid to the clinical relevance of each arterial variation [91].

Last, but not least, it is necessary to list several precise definitions for terms concerning variant vascular anatomy in order to understand their mutual differences:

- *Vas anastomoticum* = Anastomosis—a blood vessel connecting two or more vascular networks supplied by another source vessel or collected in another collecting vein.
- *Vas collaterale* = Collateral—a blood vessel forming a parallel canal supplying the same area from one source vessel or draining the same area into the same vein.
- *Vas accessorium* = Accessory vessels—a vessel supplying the same area as the proper one, but branching from the proper source vessel or from a neighbor one (a part of variant anatomy).
- *Vas aberrans* = Aberrant vessel—a vessel supplying the same area as the proper one but branching from another source vessel or from a separate network (a part of variant anatomy).

## 5. Other Parts of the Human Body

Other parts of the human body exhibit significantly fewer variations than the vascular system. We list several examples below, which we have either observed during our scientific research or encountered randomly during educational courses involving dissection of cadavers.

The synovial bursae, which are protective structures of the joints and muscle tendons, are numerous, especially in the lower limbs. Synestvedt has depicted and described 121 bursae, but only 44 of them are listed in TA [92,93,94]. The second most variable part of the human body is the muscular system, with the most variable muscle being the *musculus digastricus*. An accessory anterior belly (crossing the midline or not) has been reported in 66.7% of cases. Variations in the *musculus digastricus* have been classified into 10 different types based on variations in the *venter anterior et posterior* [95].

The variations are often related to different numbers of muscle heads (2–5 heads of the *musculus biceps brachii*), tendons (more tendons of the *musculus extensor carpi radialis longus et brevis*), accessory muscle slips and bands, rudimentary muscles (*musculus sternalis*, *musculus dorsoepitrochlearis*, *musculus epitrochleoanconeus* (previously mentioned in the Basiliensia Nomina Anatomica (BNA) in 1895, but then removed from the nomenclature)), tendinous arcades (*arcus axillaris* of Langer [49,96,97] or Struther’s canal [98]), absent *musculus palmaris longus* (missing in 4–20% of cases based on the population), absent *musculus psoas minor* (33.4–52% of cases based on the population), different numbers (3 or 4) of *intersectiones tendineae musculi recti abdominis* and their irregular arrangement, just to mention a few of the most well-known examples [22].

There exist several important literary sources, such as the classical atlas of Henle, Testut, and Le Double [99,100,101], works of Macalister and Mori [102,103], Frohse and Fränkel’s chapter concerning the muscles of the upper and lower limbs [104], Eisler’s chapter devoted to the muscles of the trunk [105], and some quite recent articles [106,107].

The bones and their variability can be easily traced on radiograms, including *fabella*, *cyamella*, *sternebrae*, *cristae phalangis* for the insertion of the *musculus flexor digitorum superficialis*, *crista musculi opponentis pollicis ossis metacarpi primi*, *spina musculi teretis majoris scapulae*, *tuberculum deltoideum claviculae*, *tuberositas interossea radii*, *tuberositas tractus iliotibialis* (tubercle of Gerdy) in limb bones. Other variabilities that are easily traced on radiograms include the *canalis arteriae vertebralis atlantis* (also referred to as *ponticulus posticus*, *foramen arcuale*, or, incorrectly, “*foramen arcuatum*”; also known by the eponym Kimmerle’s anomaly [108]) in the vertebrae. In the skull, other examples include the *foramen squamosum ossis temporalis*, persistent *foramen caecum ossis frontalis*, *foramen occipitale*, *foramen venosum* (of Vesalius), *foramen cricotaphicobuccinatorius ossis sphenoidalis* (of Hyrtl) (for details, see Hauser and Steffano [28]), or the *processus styloideus elongatus* (which causes the syndrome of Eagle [109,110]). The paranasnal sinuses and their variability is of high importance for ear–nose–throat specialists, as presented by the Anatomical Terminology Group for Sinus Disease [111] as well as maxillofacial surgeons [112].

As for internal organs, variabilities are limited to differences in shapes and divisions, such as segmentation of the lungs (*lobus veane azygos*, which should be referred to by the proper term “*lobus veane azygoi*” because the azygos is a Greek term, whose genitive should correctly be *azygoi*), bronchial tree [113], and liver based on its bloody supply [114].

As for the nervous system, the peripheral nerves are of great concern when it comes to anatomical variability, especially in the course of their trunks (e.g., the junction of the medial and lateral roots of the *nervus medianus*, the course of the upper nerves of the lumbar plexus (*nervus iliohypogastricus*, *nervus ilioinguinalis*, *nervus cutaneus femoris lateralis*, and, especially, *nervus genitofemoralis*), or the *ansa cervicalis superficialis*). A special chapter describes the *rami communicantes* (obsoletely called “anastomoses”), which appear between the cranial nerves [115] and spinal nerves. Clinically, the most interesting variabilities are connections between the *nervus medianus* and *nervus ulnaris* (e.g., “Martin-Gruber anastomosis”) for which terminology has not been unified [49].

Some structures that were previously considered to be constant have been doubted, questioned, and restudied, such as the *rami caroticotympanici arteriae carotidis internae*, which branches from the *segmentum petrosum* (C2), as mentioned above [27]. To assess the classification and incidence of anatomical variations, meta-analytic studies should be performed on each anatomical area of interest according to established guidelines, including the Preferred Reporting Items for Systematic Reviews and Meta-Analyses (PRISMA) guidelines [116] and the Anatomical Quality Assurance (AQUA) Tool [117,118]. Several studies have reviewed clinically hot topics, such as the *appendix vermiformis caeci* [119], *arteriae hepticae* [120], or *truncus coeliacus* [121].

## 6. Anomalies and Terminology

Developmental defects or congenital defects are called *anomaliae* (anomalies), their associations are termed *conjunctiones* (conjunctions), and developmental syndromes are known as *syndromata embryologica* (embryological syndromes) in the *Terminologia Embryologica* (TE) [122]. TE2 contains a list of major anomalies at the end of each chapter, which are devoted to a specific system. For example, there are 27 anomalies of the colon and appendix, including a well-known *megacolon congenitum* (also referred to as the disease of Hirschprung). However, both of these terms do not describe the unit precisely, because the enlarged segment (i.e., *megacolon*) is normal and heathy, and the affected aganglionic segment is located aborally. The eponym does not describe the anomaly’s characteristics [123,124]. This example indicates that the nomenclature for developmental defects (anomalies) should be checked and, if needed, extended and revised.

Based on the above-stated definitions, hypotheses, and facts, it is necessary to continue research concerning the prevalence and classification of anatomical variants and refine the associated nomenclature. Without unanimous and simple variant nomenclature, communication among anatomists, clinicians, students, and teachers will continue to be confused [125,126,127,128,129,130,131,132,133,134,135,136,137,138,139,140,141].

We also have a duty to publish new and extremely rare case reports or clinically relevant case reports with the highest level of anatomical knowledge and manuscript preparation as recommended by Fontaine [142] and Morrigl [143]. Last, but not least, expanding students’ knowledge and understanding of anatomical variations is a necessary and inevitable part of both undergraduate and postgraduate education [144,145].

## 7. Discussion

Variant anatomy belongs to the portfolio of knowledge and skills not only of mere anatomists, but also of clinicians working in the fields of both diagnostic techniques and therapeutic interventions. To gain and retain knowledge of the most frequent and clinically relevant anatomical variations, a simple, clear, and exactly defined nomenclature for these structures is needed. Moore [5] stated: “All anatomists are aware of variation as they encounter them annually in their dissecting laboratories.” However, for clinicians, the need of variation knowledge is influenced by their specific field. Rare variations that are not frequently encountered by anatomists can have grave consequences if neglected or overlooked in clinical practice. A unified nomenclature can simplify the description of clinically relevant anatomical variations as well as communication among clinicians when solving diagnostic and therapeutic problems. A full inventory of all known anatomical variations will never be complete. Previously undescribed variants will continue turning up, although their frequency will decrease with time. This makes it impossible for clinicians who may potentially encounter anatomical variations in their daily practice to keep up with all known variations. It is the task of anatomists to classify variations and establish an appropriate terminology with definite and detailed descriptions completed with figures and photos. It is also the task of anatomists to keep clinicians well-informed about the most relevant anatomical variants in their specific field, including the frequency and relationships to a given organ, structure, or region. Anatomists must proceed in their scientific work and prepare terminology and descriptions of variants that are suitable to be incorporated into the next revision of the *Terminologia Anatomica* or for incorporation into individual nomenclature publications concerning variant anatomy. These descriptions must be accompanied by precise anatomical definitions for each variant in question.

## 8. Conclusions

The recognition of variant anatomy and its terminology is riddled with inconsistences. The task for anatomists is to devote time and effort to two tasks: the management of anatomical variations’ classification, nomenclature, and frequency and the development of a free, accessible, detailed database, conveying comprehensive, field-related, and clinically oriented/relevant knowledge to clinicians. In contrast, clinicians are kindly asked to report the variations that they encounter in the their clinical practice, particularly those complicating diagnostic and therapeutic procedures.

## Supplementary Materials

The following are available online at https://www.mdpi.com/1010-660X/56/12/713/s1, Table S1: List of the variant anatomy terms included in the Terminologia Anatomica, Table S2: List of the variant anatomy terms included in the Terminologia Neuroanatomica, Table S3: Items in the Terminologia Anatomica [39], Terminologia Histologica [145] originated from generally used eponyms.

## Abbreviations

IFAA	International Federation of Association of Anatomists
TA	*Terminologia Anatomica*
TE	*Terminologia Embryologica*
TNA	*Terminologia Neuroanatomica*
