# Supplementary material for: Variant Anatomy and Its Terminology
Source: Medicina (Kaunas). 2020 Dec 18;56(12):713. doi: 10.3390/medicina56120713 (PMC7766054; doi:10.3390/medicina56120713)
Supplement: Supplementary file 1 [file medicina-56-00713-s001.pdf]

medicina

Review

Variant Anatomy And Its Terminology

David Kachlik [1,2], Václav Báča [2], Vladimír Musil [3] and Ivan Varga [4,*]

[1] Department of Anatomy, Second Faculty of Medicine, Charles University, V Úvalu 84, 15006 Prague, Czech Republic; david.kachlik@lfmotol.cuni.cz
[2] Department of Health Care Studies, College of Polytechnics Jihlava, Tolstého 16, 58601 Jihlava, Czech Republic; vaclav.baca@vspj.cz
[3] Centre of Scientific Information, Third Faculty of Medicine, Charles University, Ruská 87, 10000, Prague, Czech Republic; vladimir.musil@lf3.cuni.cz
[4] Institute of Histology and Embryology, Faculty of Medicine, Comenius University in Bratislava, 81372 Bratislava, Slovakia; ivan.varga@fmed.uniba.sk
* Correspondence:Correspondence: ivan.varga@fmed.uniba.sk; Tel.: +421 2 90 119 547 (I.V.)

Received: date; Accepted: date; Published: 21 December 2020

Supplementary Material

Table S1. List of the variant anatomy terms included in the *Terminologia Anatomica* [39].

Identification number	Latin term
A01.2.05.004	(Foveola coccygea)
A01.2.05.009	(Trigonum lumbale inferius)
A01.2.05.010	(Trigonum lumbale superius)
A02.1.00.043	(Os suturale)
A02.1.00.063	(Torus palatinus)
A02.1.03.007	(Sutura frontalis persistens; Sutura metopica)
A02.1.03.024	(Spina trochlearis)
A02.1.04.013	(Os interparietale)
A02.1.04.023	(Crista occipitalis externa)
A02.1.04.030	(Crista occipitalis interna)
A02.1.04.035	(Processus paramastoideus)
A02.1.05.008	(Processus clinoideus medius)
A02.1.05.037	(Foramen venosum)
A02.1.05.039	(Foramen petrosum)
A02.1.06.070	(Spina suprameatica; Spina suprameatalis)
A02.1.12.031	(Os incisivum; Premaxilla)
A02.1.12.032	(Sutura incisiva)
A02.1.14.008	(Tuberculum marginale) ossis zygomatici
A02.1.15.004	(Symphysis mandibulae)
A02.1.15.013	(Torus mandibularis)
A02.1.15.026	(Tuberositas masseterica)
A02.1.15.027	(Tuberositas pterygoidea)
A02.2.02.110	(Canalis arteriae vertebralis)
A02.3.02.013	(Costa cervicalis; Costa colli)

A02.3.02.020	(Costa lumbalis)
A02.3.03.009	(Ossa suprasternalia)
A02.4.04.017	(Processus supracondylaris)
A02.4.08.002	(Os centrale)
A02.5.01.312	(Tuberculum obturatorium posterius)
A02.5.04.008	(Trochanter tertius)
A02.5.10.021	(Os trigonum)
A03.0.00.024	(Caput articulare)
A03.1.02.012	(Sutura squamomastoidea)
A03.1.05.006	(Synchondrosis intraoccipitalis posterior)
A03.1.05.007	(Synchondrosis intraoccipitalis anterior)
A03.1.08.003	(Lig. atlantooccipitale anterius)
A03.3.02.007	(Synchondrosis manubriosternalis)
A03.5.01.004	(Lig. transversum scapulae inferius)
A03.5.03.003	(Discus articularis) articulationis acromioclavicularis
A04.2.01.007	(M. scalenus minimus)
A04.2.04.008	(M. levator glandulae thyroideae)
A04.3.01.005	(M. transversus nuchae)
A04.3.02.211	(Mm. rotatores lumborum)
A04.4.01.001	(M. sternalis)
A04.5.04.009	(Arcus tendineus musculi levatoris ani)
A04.7.02.005	(M. psoas minor)
A04.7.02.064	(M. abductor metatarsi quinti)
A04.7.02.065	(M. opponens digiti minimi)
A04.8.03.003	(Bursa subcutanea acromialis)
A04.8.03.006	(Bursa musculi coracobrachialis)
A04.8.03.012	(Bursa intratendinea olecrani)
A04.8.03.015	(Bursa cubitalis interossea)
A04.8.05.011	(Bursa iliopectinea)
A04.8.05.015	(Bursa subfascialis prepatellaris)
A04.8.05.016	(Bursa subtendinea prepatellaris)
A05.1.03.071	(Tuberculum anomale) dentis
A05.1.03.078	(Distema)
A05.1.04.021	(Ductus thyroglossalis)
A05.2.01.006	(Plica triangularis faucium)
A05.2.01.008	(Plica semilunaris faucium)
A05.2.01.013	(Fissura tonsillaris) tonsillae palatinae; (Fissura intratonsillaris)
A05.3.01.010	(Bursa pharyngealis)
A05.6.04.003	(Diverticulum ilei)
A05.7.02.010	(Fascia precaecocolica)
A05.9.01.018	(Pancreas accessorium)
A06.1.02.033	(Ductus incisivus)

A06.2.02.012	(Foramen thyroideum) cartilaginis thyroideae
A06.2.04.018	(Cartilago sesamoidea) laryngis
A06.2.08.005	(M. ceratocricoideus)
A08.1.05.008	(Typus ampullaris) pelvis renalis
A09.1.06.002	(Ductus deferens vestigialis)
A09.2.03.015	(Ductus paraurethrales)
A09.3.02.008	(Ductulus aberrans superior)
A09.3.02.009	(Ductulus aberrans inferior)
A09.3.02.011	(Appendix epididymidis)
A09.3.03.001	(Paradidymis)
A09.3.04.006	(Vestigium processus vaginalis peritonei)
A09.3.08.015	(Lobus medius prostatae)
A09.4.02.023	(Valvula fossae navicularis)
A10.1.02.011	(Mesocolon ascendens)
A10.1.02.012	(Mesocolon descendens)
A10.1.02.106	(Lig. hepatocolicum)
A10.1.02.205	(Lig. gastrocolicum)
A10.1.02.414	(Plica paraduodenalis)
A10.1.02.415	(Recesssus paraduodenalis)
A10.1.02.416	(Recessus retroduodenalis)
A11.3.00.004	(Lobus pyramidalis) glandulae thyroideae
A12.1.01.007	(Foramen ovale cordis)
A12.2.01.202	(Ductus arteriosus)
A12.2.03.111	(R. posterolateralis dexter) arteriae coronariae dextrae
A12.2.03.212	(R. nodi sinuatrialis) arteriae coronariae sinistrae
A12.2.03.213	(R. nodi atrioventricularis) arteriae coronariae sinistrae
A12.2.04.002	(Isthmus aortae)
A12.2.04.005	(A. thyroidea ima)
A12.2.05.014	(Truncus linguofacialis)
A12.2.05.035	(R. meningeus) arteriae occipitalis
A12.2.05.041	(R. stapedius arteriae auricularis posterioris)
A12.2.06.020	(A. uncalis)
A12.2.07.003	(Rr. choroidei ventriculi tertii) arteriae choroideae anterioris
A12.2.07.014	(Rr. uncales) arteriae choroideae anterioris
A12.2.07.016	(Rr. tuberis cinerei) arteriae choroideae anterioris
A12.2.07.017	(Rr. nucleorum hypothalami) arteriae choroideae anterioris
A12.2.07.042	(Rr. paracentrales) arteriae pericallosae
A12.2.08.032	(Rr. bronchiales) arteriae thoracicae internae
A12.2.08.033	(Rr. tracheales) arteriae thoracicae internae
A12.2.08.038	(R. costalis lateralis) arteriae thoracicae internae
A12.2.08.058	(A. dorsalis scapulae)
A12.2.09.019	(A. brachialis superficialis)

A12.2.12.017	(A. supraduodenalis)
A12.2.15.032	(A. azygos vaginae) ♀
A12.2.16.006	(A. obturatoria accessoria)
A12.2.16.044	(A. recurrens tibialis posterior)
A12.2.16.051	(A. arcuata)
A12.2.16.070	(Arcus plantaris superficialis)
A12.3.01.016	(Vv. atriales sinistrae)
A12.3.01.017	(Vv. ventriculares sinistrae)
A12.3.02.110	Pars intralobaris (intersegmentalis) v. posterioris pulmonalis dx. sup.
A12.3.04.015	(V. vertebralis accessoria)
A12.3.08.017	(V. cephalica accessoria)
A13.1.02.006	(Lobuli thymici accessorii)
A13.2.01.012	(Facies pancreatica splenis)
A13.2.01.022	(Splen accessorius)
A13.3.02.008	(Nodus lymphoideus ligamenti arteriosi)
A13.3.02.009	(Nodus lymphoideus arcus venae azygos)
A13.3.03.017	(Anulus lymphaticus cardiae)
A13.3.03.020	(Nodus lymphoideus suprapyloricus)
A13.3.03.021	(Nodi lymphoidei subpylorici)
A13.3.03.022	(Nodi lymphoidei retropylorici)
A13.3.04.013	(Nodus lymphoideus lacunaris medialis)
A13.3.04.014	(Nodus lymphoideus lacunaris intermedius)
A13.3.04.015	(Nodus lymphoideus lacunaris lateralis)
A13.3.05.008	(Nodus lymphoideus inguinalis profundus proximalis)
A13.3.05.009	(Nodus lymphoideus inguinalis profundus intermedius)
A13.3.05.014	(Nodus lymphoideus tibialis anterior)
A13.3.05.015	(Nodus lymphoideus tibialis posterior)
A13.3.05.016	(Nodus lymphoideus fibularis)
A14.1.01.109	(Spatium subdurale)
A14.1.01.110	(Spatium epidurale; Spatium extradurale)
A14.2.01.112	(R. lingualis) nervi facialis
A14.2.02.031	(Nn. phrenici accessorii)
A14.3.01.019	(Ganglion cervicale inferius)
A15.2.03.036	(Membrana pupillaris)
A15.2.06.009	(A. hyaloidea)
A15.2.07.061	(Glandulae lacrimales accessoriae)
A15.3.01.020	(Tuberculum auriculare)
A15.3.01.021	(Apex auriculae)
A15.3.01.023	(Tuberculum supratragicum)
A15.3.01.040	(M. incisurae terminalis)
A16.0.02.003	(Mamma accessoria)

Table S2. List of the variant anatomy terms included in the *Terminologia Neuroanatomica* [38] (compared to *Terminologia Anatomica* [39]).

TNA 2017	TA 1998
**Systema nervosum centrale**	
(Spatium epidurale)	(Spatium epidurale; Spatium extradurale)
(Spatium subdurale)	(Spatium subdurale)
(Arteria hypoglossa persistens)	-
(Arteria trigemina persistens)	-
(Arteria otica persistens)	-
(Rami choroidei ventriculi tertii) arteriae choroideae anterioris	(Rr. choroidei ventriculi tertii) arteriae choroideae anterioris
(Rami uncales) arteriae choroideae anterioris	(Rr. uncales) arteriae choroideae anterioris
(Rami tuberis cinerei) arteriae choroideae anterioris	(Rr. tuberis cinerei) arteriae choroideae anterioris
(Rami nucleorum hypothalami) arteriae choroideae anterioris	(Rr. nucleorum hypothalami) arteriae choroideae anterioris
(Arteria subcallosa)	-
(Rami paracentrales) arteriae cerebri anterioris	(Rr. paracentrales) arteriae pericallosae
(Arteria proatlantica)	-
(Ramus meningeus) arteriae cerebelli inferioris posterioris	-
(Sinus occipitalis obliquus)	-
(Plexus venosus falcis)	-
(Sinus intercavernosus inferior)	-
(Cellula rotiformis)	-
**Systema nervosum periphericum**	
(Nervus ethmoidalis posterior)	-
(Rami ganglionares sublinguales)	-
(Ramus lingualis) nervi facialis	(R. lingualis) n. facialis
(Radix posterior nervi spinalis C1)	-
(Ganglion spinale nervi spinalis C1)	-
(Ramus cutaneus posterior) nervi cervicalis quinti	-
(Nervus phrenicus accessorius)	(Nn. phrenici accessorii)
(Radix lateralis nervi ulnaris)	-
(Ramus communicans ulnaris) nervi mediani	-
(Nervus intercostobrachialis accessorius)	-
(Ramus communicans iliohypogastricus) nervi thoracici duodecimi	-
(Nervus obturatorius accessorius)	-
(Ganglion vertebrale)	-
(Radix sympathica ganglii sublingualis)	-
(Ganglion sublinguale)	-

Organa sensuum	
(Arteria cilioretinalis)	-
(Membrana pupillaris)	(Membrana pupillaris)
(Arteria hyaloidea)	(A. hyaloidea)
(Glandula lacrimalis accessoria)	(Glandulae lacrimales accessoriae)
(Tuberculum auriculare)	(Tuberculum auriculare)
(Apex auriculae)	(Apex auriculae)
(Tuberculum supratragicum)	(Tuberculum supratragicum)
(Musculus incisurae terminalis)	-
(Ramus vestibularis anterior)	-

Table S3. Items in the *Terminologia Anatomica* [39], *Terminologia Histologica* [145] originated from generally used eponyms.

Eponym	Identification number	Latin term	English equivalent
Jan Evangelista Purkyně (Purkinje)	A14.1.07.404	Stratum purkinjense	Purkinje cell layer
Ammon (Ra)	A14.1.09.327	Hippocampus proprius; Cornu ammonis	Hippocampus proper; Ammon´s horn
Camillo Golgi	H1.00.01.3.01046	Complexus golgiensis; Apparatus golgiensis	Golgi complex; Golgi apparatus
Schwann	H2.00.06.2.02003	Schwannocytus; Neurolemmocytus	Schwann cell; Neurolemmocyte
Paneth	H3.04.03.0.00017	Cellula panethensis; Exocrinocytus cum granulis acidophilicus	Paneth cell

© 2020 by the authors. Licensee MDPI, Basel, Switzerland. This article is an open access article distributed under the terms and conditions of the Creative Commons Attribution (CC BY) license (http://creativecommons.org/licenses/by/4.0/).
